# Resistance to hormone therapy in breast cancer cells promotes autophagy and EGFR signaling pathway

**DOI:** 10.1152/ajpcell.00199.2023

**Published:** 2023-08-14

**Authors:** Konstantinos E. Siatis, Efstathia Giannopoulou, Dimitra Manou, Panagiotis Sarantis, Michalis V. Karamouzis, Sofia Raftopoulou, Konstantinos Fasseas, Fatimah Mohammed Alzahrani, Haralabos P. Kalofonos, Achilleas D. Theocharis

**Affiliations:** ^1^Biochemistry, Biochemical Analysis and Matrix Pathobiology Research Group, Laboratory of Biochemistry, Department of Chemistry, University of Patras, Rio, Greece; ^2^Clinical Oncology Laboratory, Division of Oncology, Department of Medicine, University of Patras, Rio, Greece; ^3^Molecular Oncology Unit, Department of Biological Chemistry, Medical School, National and Kapodistrian University of Athens, Athens, Greece; ^4^Electron Microscopy Laboratory, Faculty of Crop Production, Agricultural University of Athens, Athens, Greece; ^5^Department of Chemistry, College of Science, Princess Nourah bint Abdulrahman University, Riyadh, Saudi Arabia

**Keywords:** autophagy, breast cancer, EGFR signaling, endocrine resistance, ERα

## Abstract

Breast cancer is the leading cause of cancer deaths for women worldwide. Endocrine therapies represent the cornerstone for hormone-dependent breast cancer treatment. However, in many cases, endocrine resistance is induced with poor prognosis for patients. In the current study, we have developed MCF-7 cell lines resistant to fulvestrant (MCF-7Fulv) and tamoxifen (MCF-7Tam) aiming at investigating mechanisms underlying resistance. Both resistant cell lines exerted lower proliferation capacity in two-dimensional (2-D) cultures but retain estrogen receptor α (ERα) expression and proliferate independent of the presence of estrogens. The established cell lines tend to be more aggressive exhibiting advanced capacity to form colonies, increased expression of epidermal growth factor receptor (EGFR), human epidermal growth factor receptor 2 (HER2), and heterodimerization of ERBB family receptors and activation of EGFR downstream pathways like MEK/ERK1/2 and PI3K/AKT. Tyrosine kinase inhibitors tested against resistant MCF-7Fulv and MCF-7Tam cells showed moderate efficacy to inhibit cell proliferation, except for lapatinib, which concomitantly inhibits both EGFR and HER2 receptors and strongly reduced cell proliferation. Furthermore, increased autophagy was observed in resistant MCF-7Fulv and MCF-7Tam cells as shown by the presence of autophagosomes and increased Beclin-1 levels. The increased autophagy in resistant cells is not associated with increased apoptosis, suggesting a cytoprotective role for autophagy that may favor cells’ survival and aggressiveness. Thus, by exploiting those underlying mechanisms, new targets could be established to overcome endocrine resistance.

**NEW & NOTEWORTHY** The development of resistance to hormone therapy caused by both fulvestrant and tamoxifen promotes autophagy with concomitant apoptosis evasion, rendering cells capable of surviving and growing. The fact that resistance also triggers ERBB family signaling pathways, which are poorly inhibited by tyrosine kinase inhibitors might attribute to cells’ aggressiveness. It is obvious that the development of endocrine therapy resistance involves a complex interplay between deregulated ERBB signaling and autophagy that may be considered in clinical practice.

## Graphical abstract

**Figure:**
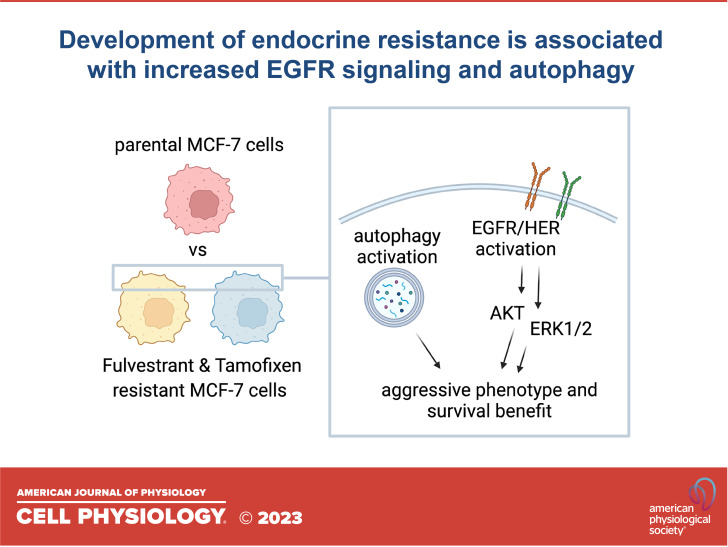

## INTRODUCTION

Breast cancer is considered the most frequent cause of female mortality in less developed countries, whereas in the more developed ones, it is responsible as the second cause of cancer death (1). Historically, breast cancer was classified according to the expression of the estrogen receptor (ER), progesterone receptor (PR), and human epidermal growth factor receptor 2 (HER2). Further advancements on the characterization of breast cancer led to their classification into five intrinsic subtypes (luminal A, luminal B, HER2-enriched, and basal normal-like) according to the expression of a panel of 50 genes (2). Although hormone-dependent estrogen receptor-positive (ER^+^) luminal A and luminal B breast cancer subtypes are associated with better prognosis, they present very often with annual relapse or long-term resistance to endocrine treatment options (3). Endocrine/hormone therapies represent the mainstay in the treatment of hormone-dependent breast cancer and act either by modulating or disrupting estrogen production and the presence or function of ERs (4). Tamoxifen (Tam) and fulvestrant (Fulv) are major representatives of selective ER modulators (SERMs) and selective ER degraders/downregulators (SERDs), respectively, belonging thus to routine therapy of patients with ER^+^ breast cancer. Aromatase inhibitors (AIs), such as exemestane, pose another therapeutic option by decreasing estrogen levels (5, 6). Although estrogen-based therapies have changed the history of hormone-dependent breast cancer, many tumors are drug-resistant either de novo or acquired (3, 7).

Resistance to hormone therapies in breast cancer cells can be addressed either by loss or retention of ER expression rendering breast cancer cells estrogen dependent or independent (8). Endocrine resistance usually causes evolutionary molecular alterations that may result in the activation of alternative cell signaling pathways such as multidirectional crosstalk among growth factors, overexpression of them or their receptors like epidermal growth factor receptor (EGFR), HER2, and insulin growth factor 1 receptor (IGF1R), and even activation of downstream signaling (9, 10).

On the other hand, autophagy is a mechanism involved not only in cell homeostasis but also in cancer cell biology. Autophagy is a housekeeping mechanism for degrading the damaged or unnecessary cell structures and organelles under stress conditions to promote cell survival (11). However, when the stress severity or duration increases, autophagy may lead to cell death. The imbalance between survival and death is a key characteristic of cancer cells. Autophagy has a dual function as it can be tumor suppressive at early stages but tumor promotional in established diseases. Tumor cells may use autophagy to survive in environments with poor nutrients, or they can die because of autophagy (11). In breast cancer cells, autophagy tends to induce metastasis by retaining and expanding cell survival and might render cells to enter a dormant state if they cannot establish stable contact with the extracellular matrix in the new environment (12). During autophagy, the autophagic cargo is surrounded by a double membrane creating the autophagosomes. These double-membrane vesicles are fused with the lysosomes generating the autolysosomes where the autophagic cargo is degraded. During autophagy, several proteins are activated including Beclin-1. Bcl-2 phosphorylation by stress-activated c-Jun N-terminal protein kinase 1 (JNK1) as well as Beclin-1 phosphorylation by kinases including death-associated protein kinase (DAPK) results in the dissociation of Beclin-1 from Bcl-2 and autophagy activation (13, 14). Beclin-1 is a very important partner for the localization of autophagic proteins (11).

The current study aimed to develop cells resistant to endocrine therapy, to study underlying mechanisms of acquired resistance and shed light on the progression of hormone-dependent breast cancer. We found that autophagy might be a mechanism that favors resistant cells’ growth and survival accompanied by a simultaneous overexpression and activation of tyrosine kinase receptors, such as EGFR and HER. This model might imply a more aggressive behavior concerning cells’ proliferation and breast cancer evolution.

## MATERIALS AND METHODS

### Cell Culture and Reagents

Hormone-dependent breast cancer cell line MCF-7 was purchased from the American Type Culture Collection (ATCC, LGC Standards, Wesel, Germany) and cultured in phenol red-free medium RPMI (rf-RPMI) with 10% charcoal-stripped serum (CSS) supplemented with 100 µg/mL penicillin G/streptomycin, 50 µg/mL gentamycin, 0.01 mg/mL insulin, and 5 nM exogenous estradiol (E_2_) (4, 6, 15). The development of MCF-7 cells resistant to fulvestrant (Fulv) and tamoxifen (Tam) was achieved after the long-term culture of cells with gradually increasing concentrations of each agent alone (16). MCF-7 cells resistant to Fulv or Tam will be named hereafter MCF-7/Fulv and MCF-7/Tam, respectively. MCF-7/Fulv and MCF-7/Tam cells were maintained in cultures containing 40 nM Fulv or 40 nM Tam, respectively, along with the presence of 5 nM exogenous estradiol (E_2_) unless otherwise stated. All culture mediums and supplements were purchased from Biochrom (Berlin, Germany). E_2_, Fulv, Tam, and insulin were purchased from Sigma-Aldrich (Sigma-Aldrich, Inc.). Epidermal growth factor (EGF) 10 ng/mL (Peprotech), Exemestane, (Aromasin, Pfizer), Cetuximab 50 μg/mL (Erbitux Bristol-Myers Squibb), Trastuzumab 50 μg/mL (Herceptin Roche), Gefitinib 20 μM (Iressa AstraZeneca), Lapatinib 20 μM (Tyverb GlaxoSmithKline), and Erlotinib 11 μM (Tarceva Roche) were used, respectively (17–19). Cells were cultured at 37°C, 5% CO_2_, and 100% humidity.

### Cell Proliferation Assay

Cells were seeded in 48-well culture plates (10,000 cells/well). After the appropriate treatment, a volume equal to 1/10 of the volume in each well of the 5 mg/mL solution of 3-[4,5-dimethylthiazol-2-yl]-2,5-dimethyltetrazolium bromide (MTT) (20) in phosphate buffer saline (PBS) was added, and the cells were incubated at 37°C, 5% CO_2_, and 100% humidity for 2 h. After that, the medium was removed, and 100 μL of acidified isopropanol (0.33 mL HCl in 100 mL isopropanol) was added in each well. The suspension was transferred to a 96-well plate and measured in a spectrophotometer (Tecan Sunrise, Magellan 2, Grodig, Austria) at 570 nm with a correction at 620 nm.

### Colony Formation Assay

To culture MCF-7, MCF-7/Fulv, and MCF-7/Tam cells in three-dimensional (3-D) conditions, a soft agar assay was applied using a standard procedure. Briefly, in a 12-well plate, a bottom layer consisting of 0.7% agar in 1 mL rf-RPMI with 10% CSS was first allowed to solidify in each well. The appropriate number of cells (75 × 10^3^ cells/well) was mixed with 1 mL rf-RPMI with 10% CSS that contains 0.5% agar and applied on the top of the bottom agar media layer. Each well was further supplemented with 1 mL fresh rf-RPMI with 10% CSS once a week. After 10–15 days of incubation at 37°C, 5% CO_2_, and 100% humidity, cells were stained with crystal violet and visualized in an inverted microscope (Axiovert 40 CFL, AxioCam ERc, Zeiss, Germany) at a magnitude of ×4 or ×10.

### Transmission Electron Microscopy

MCF-7, MCF-7/Fulv, and MCF-7/Tam cells were cultured in a six-well plate at a density of 1 × 10^5^ cells/well for 24 h and then a standard procedure was applied (21). Briefly, cells were washed once with PBS and then fixed with 2.5% glutaraldehyde at 4°C for 2 h. After washing with PBS, cells were dehydrated by using a gradient of ethanol and finally incubated with 100% acetone for 15 min and embedded in SPURR resin. Ultrathin sections were taken and stained with uranyl acetate and lead citrate, and images were captured by using a JEOL 100S transmission electron microscope equipped with an Olympus MegaView G2 camera.

### RNA Isolation and Real-Time qPCR

Total RNA was isolated from MCF-7, MCF-7/Fulv, and MCF-7/Tam cells using RNeasy Plus Mini Kit (Qiagen, Germany). Isolated RNA was quantified by measuring its absorbance at 260 nm with a spectrophotometer nanodrop 1000 (Thermo Fisher Scientific Inc.). PrimeScript 1st strand cDNA synthesis kit perfect real-time (Takara Bio Inc., Japan) was used to reverse transcribe total RNA. Gene amplification was performed by using KAPA Taq ReadyMix DNA Polymerase (KAPABIOSYSTEMS). Quantitative RT-PCR analysis was performed in a 20-μL reaction mixture, according to the standard protocol in a Rotor Gene Q equipment (Qiagen). All reactions were performed in triplicates, and a standard curve was always included for each pair of primers for assay validation. In addition, a melting curve analysis was always performed for detecting the SYBR Green-based objective amplicon. To provide quantification, the point of product accumulation in the early logarithmic phase of the amplification plot was defined by assigning a fluorescence threshold above the background, defined as the threshold cycle (Ct) number. ΔΔCt method was used to calculate the relative expression of different gene transcripts. The Ct of *ERα*, *EGFR*, and *HER2* was normalized to the Ct of the housekeeping gene *GAPDH*. Fold changes (arbitrary units) were determined as 2^−ΔΔCt^. The utilized primers for *ERα*, *EGFR*, *HER2*, and *GAPDH* have been described previously (22). All primers were purchased from Eurofins Genomics (Ebersberg, Germany).

### Apoptosis Assay

Cells were plated at 3 × 10^4^ cells/well in 24-well plates and cultured with Fulv and Tam as described previously. The tested agents were added to cells at the indicated concentration. After 48 h of incubation, cells were washed twice with PBS, trypsinized for 4 min, and centrifuged for 4 min at 166 *g*. Apoptotic and necrotic cells were detected using the Muse Annexin V & Dead Cell kit, according to the manufacturer’s instructions (Merck-Millipore, Germany). Briefly, cells were resuspended in a medium with 1% FBS, and 100 μL from cell suspension was mixed with 100 μL of Muse Annexin V & Dead Cell reagent for 20 min, protected from light at room temperature, and analyzed by Muse Cell Analyzer, according to the standard protocol (Muse software, Merck-Millipore, Germany). The assay application using annexin V (An) and a dead cell marker (DCM) distinguishes four populations; the viable (An−/DCM−), the early apoptotic (An+/DCM−), the late apoptotic (An+/DCM+), and the necrotic (An−/DCM+) cells. The number of total apoptotic cells is the sum of early and late apoptotic cells.

### Proximity Ligation Assay

We applied proximity ligation assay (PLA) on breast cancer cells to visualize endogenous protein interactions as described previously (23). Briefly, cells were grown in 4-well coverslips. After 24 h of incubation, cells were fixed with 4% paraformaldehyde in PBS for 20 min at room temperature and permeabilized for 10 min, and PLA (Sigma-Aldrich) was conducted as described in the Duolink protocol using appropriate sets of primary antibodies (EGFR Santa Cruz sc-373746, HER2 Santa Cruz sc-33684, HER3 Cell Signaling #12708, and HER4 Santa Cruz sc-283). Nuclei staining was performed by incubating cells with TOPRO3 (1:1,000 in PBS) for 2 min and then the coverslips were mounted with MOWIOL (Sigma-Aldrich). Fluorescence images were taken by using a Zeiss Axiovert microscope (Carl Zeiss Microscopy). Representative images were captured from at least 10 randomly selected fields for each coverslip.

### Immunoprecipitation and Immunoblotting

Cells were plated at a density of 1 × 10^6^ cells per Petri dish and cultured for 48 h as previously described. Cells were lysed by adding lysis buffer (50 mM Tris-HCl pH 7.5, 150 mM NaCl, 5 mM EDTA, 1% Triton, 10% glycerol, 1 mM phenylmethyl-sulphonyl-fluoride, 2 mM Na-orthovanadate, and 10 mM leupeptin), and protein concentration was estimated by Bradford assay. Part of cell lysates (1 mg of total protein) was used for immunoprecipitation. Cell lysates were incubated with a mouse monoclonal anti-Bcl2-antibody (sc-509, 1:200, Santa Cruz) overnight at 4°C, under continuous agitation. Then, 50 μL of protein-A sepharose beads (Sigma, Amersham) were added to each sample and incubated for 4 h, at 4°C, under continuous agitation. After centrifugation, the precipitates were washed twice with ice-cold lysis buffer. The sepharose beads were dissolved in 50 μL 2× sample buffer (0.5 M Tris-HCl pH 6.8, 20% glycerol, 2% SDS, 2% bromophenol blue, and 10% β-mercaptoethanol), boiled for 5 min at 95°C, and then subjected to SDS-PAGE and immunoblotting. Aliquots of cell lysates were also analyzed by SDS-PAGE and immunoblotting (24, 25). A goat polyclonal anti-Beclin-1 (1:500, sc-10086 Santa Cruz Biotechnology Inc.), a rabbit monoclonal anti-Bcl-2 (1:500, sc-509 Santa Cruz Biotechnology Inc.), a mouse monoclonal anti-actin antibody (1:1,000, MAB1501 Chemicon, Millipore, Temecula, CA), a rabbit monoclonal anti-EGFR (1:5,000, 04-338 Upstate, Chemicon, Millipore, Temecula, CA), a rabbit monoclonal anti-pEGFR (Tyr1173) (1:500, 05-1004 Millipore, Temecula, CA), a rabbit polyclonal anti-ERK1/2 (1:1,000, 9102 Cell Signaling Technology, Leiden, The Netherlands), a rabbit polyclonal anti-pERK1/2 (1:1,000, 9101 Cell Signaling Technology, Leiden, The Netherlands), a goat polyclonal anti-AKT (1:500, sc-1618, Santa Cruz Biotechnology Inc.), a rabbit polyclonal anti-pAKT (1:2,000, 4060 Cell Signaling Technology, Leiden, The Netherlands), and a mouse monoclonal anti-ERα (1:200, NCL-L-ER-6F11, Novocastra, Leica) were used. The immunoreactive proteins were detected by chemiluminescence using horseradish peroxidase substrate SuperSignal (Pierce, Rockford, IL). The density of immunoreactive bands was analyzed using ImageJ Software where background was subtracted followed by normalization to the loading control obtained from the same gel (actin), and a percentage relative to the control MCF-7 cells was obtained.

### Statistical Analysis

The experiments were performed in triplicates, and the results are expressed as mean ± standard deviation (SD). At least three independent biological samples have been analyzed in each experimental set. Statistically significant differences were examined using the analysis of variance (two-way ANOVA) test and were considered statistically significant at the level of *P* ≤ 0.05. GraphPad Prism 8.2.1. software was used for the statistical analysis and construction of the graphs.

## RESULTS

### Establishment of Breast Cancer Cell Lines Resistant to Fulvestrant and Tamoxifen

To evaluate the effect of Fulv and Tam on MCF-7 cells and estimate IC_50_ values of parental MCF-7 cells, they were cultured in RPMI without phenol red supplemented with 10% CSS in the presence of increasing concentrations of Fulv and Tam for 48 h, and MTT assay was performed. Estimated IC_50_ values of parental MCF-7 cells to Fulv and Tam were 56 nM and 58 nM, respectively (Table 1). Then, we went to create MCF-7 cells resistant to Fulv and Tam. After several passages with increased dose escalation of each agent, Fulv-resistant MCF-7 cells (MCF-7/Fulv) and Tam-resistant MCF-7 cells (MCF-7/Tam) were established and their IC_50_ values were determined as described previously. Indeed, IC_50_ values were dramatically changed by more than 100-fold before and after the development of resistance as shown in Table 1.

**Table 1.** IC_50_ values before and after the development of resistance to hormone therapy

Cells	IC_50_ Fulv	IC_50_Tam
MCF-7	56 nM	58 nM
MCF-7/Fulv	>100 μΜ	
MCF-7/Tam		>100 μΜ

Fulv, fulvestrant; MCF-7/Fulv, MCF-7 cells resistant to Fulv; MCF-7/Tam, MCF-7 cells resistant to Tam; Tam, Tamoxifen.

### Effect of Fulvestrant, Tamoxifen, and Exemestane on Cell Proliferation

**Figure 1.**
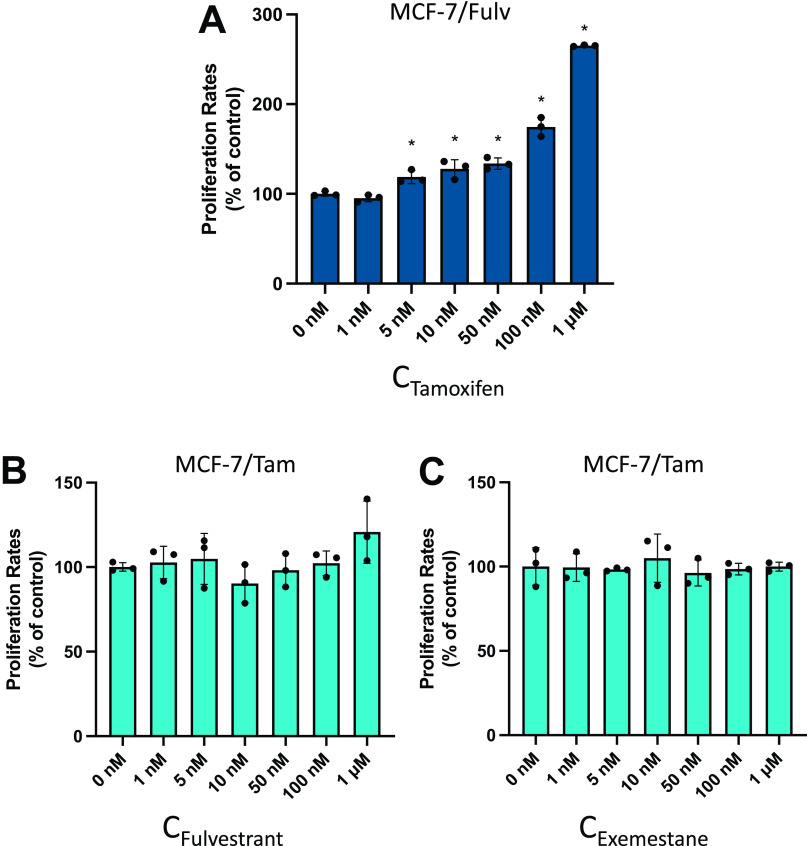
Effect on cell proliferation of tamoxifen (Tam) on MCF-7/Fulv (*A*), fulvestrant (Fulv) on MCF-7/Tam (*B*), and Exemestane on MCF-7/Tam (*C*) as measured by MTT assay. Cells were seeded in 48-well culture plates (10,000 cells/well) and cultured in RPMI without phenol red supplemented with 10% charcoal-stripped serum (CSS) in the presence of increasing concentrations of respective agents for 48 h. Results are expressed as mean ± SD of % change to control untreated cells of three independent experiments. *Statistically significant differences (*P* ≤ 0.05) compared with control untreated cells. MTT, 3-[4,5-dimethylthiazol-2-yl]-2,5-dimethyltetrazolium bromide.

After the establishment of resistant cell lines MCF-7/Fulv and MCF-7/Tam, an MTT assay was performed to estimate whether the addition of Tam on MCF-7/Fulv, Fulv on MCF-7/Tam, and Exemestane on MCF-7/Tam had any effect on cell proliferation (Fig. 1). During clinical practice, Fulv or aromatase inhibitors like Exemestane are used in patients with either metastatic breast cancer, or previously treated with Tam. Unfortunately, the outcome is ambiguous, since there is not always a survival advantage for the patients (9, 26–29). We found that the addition of Tam on MCF-7/Fulv-induced cell proliferation in a statistically significant manner (Fig. 1*A*). Similar effects of induction of cell proliferation have also been mentioned previously (30). On the contrary, the addition of Fulv and Exemestane did not affect the proliferation of MCF-7/Tam (Fig. 1, *B* and *C*).

### Fulvestrant- and Tamoxifen-Resistant MCF-7 Cells Exhibit Increased Tumorigenic Potential

**Figure 2.**
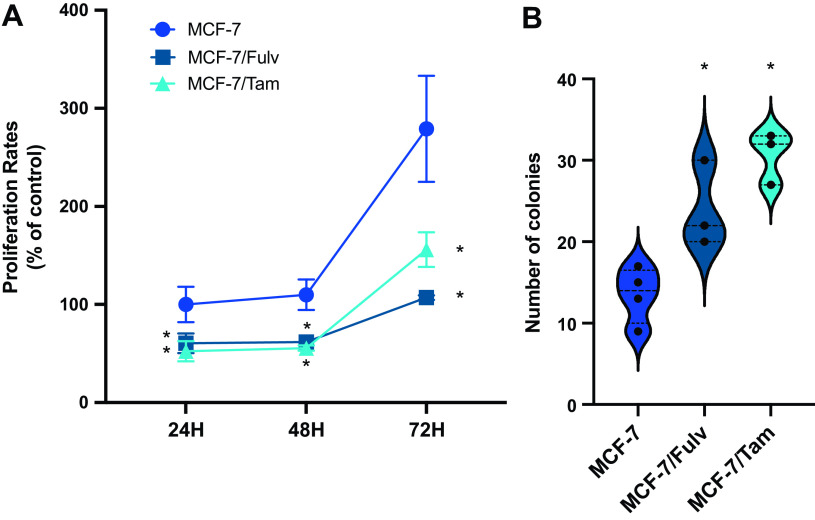
*A:* cell proliferation of MCF-7, MCF-7 cells resistant to fulvestrant (MCF-7/Fulv), and MCF-7 cells resistant to tamoxifen (MCF-7/Tam) at specific time points measured by MTT assay. Cells were seeded in 48-well culture plates (10,000 cells/well) and cultured in RPMI without phenol red supplemented with 10% charcoal-stripped serum (CSS) for 24, 48, and 72 h. Results are expressed as mean ± SD of % change to control parental MCF-7 cells at 24 h of at least three independent experiments. *Statistically significant differences (*P* ≤ 0.05) compared with control cells at the respective time points. *B*: quantification of colonies per optical field measured in colony formation assay of MCF-7, MCF-7/Fulv, and MCF-7/Tam. Results are expressed as mean ± SD of % change to control untreated cells of at least three independent experiments. *Statistically significant differences (*P* ≤ 0.05) compared with control cells. MTT, 3-[4,5-dimethylthiazol-2-yl]-2,5-dimethyltetrazolium bromide.

The proliferation rate of resistant cell lines was evaluated on two-dimensional (2-D) cell cultures at various time points. Both MCF-7/Fulv and MCF-7/Tam had lower proliferation rates compared with parental MCF-7 cells (Fig. 2*A*). This behavior may underline lower metabolic rates for resistant cell lines to use their metabolic potential in other cell functions like migration and survival under stress factors. Similar findings have been described in glioblastoma multiform cells after the acquisition of resistance toward tyrosine kinase inhibitors (31). Colony formation experiments performed in 3-D cultures revealed a survival and proliferating advantage for MCF-7/Fulv and MCF-7/Tam compared with parental MCF-7 (Fig. 2*B*). Usually, MCF-7 are difficult to survive and proliferate in soft agar, since they are considered to be less aggressive compared with other breast cancer cell lines (32). In contrast to parental MCF-7 cells, MCF-7/Fulv and MCF-7/Tam exerted a more aggressive phenotype by establishing more colonies in 3-D cultures compared with MCF-7.

### Cell Proliferation of Resistant Cell Lines Is Independent of ERα Expression and Estrogens

**Figure 3.**
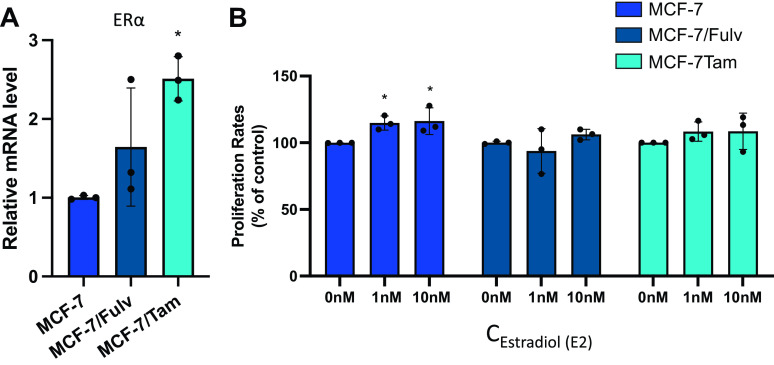
*A*: mRNA levels of estrogen receptor α (ERα) in MCF-7, MCF-7 cells resistant to fulvestrant (MCF-7/Fulv), and MCF-7 cells resistant to tamoxifen (MCF-7/Tam) measured by RT-qPCR. Results are expressed as mean ± SD of relative mRNA level of three independent experiments. *Statistically significant differences (*P* ≤ 0.05) compared with parental MCF-7 cells. *B*: effect of estradiol on cell proliferation in MCF-7, MCF-7/Fulv, and MCF-7/Tam estimated by MTT assay. Cells were seeded in 48-well culture plates (10,000 cells/well) and cultured in RPMI without phenol red supplemented with 10% charcoal-stripped serum (CSS) for 48 h. Results are expressed as mean ± SD of % change to control untreated cells of three independent experiments. *Statistically significant differences (*P* ≤ 0.05) compared with control untreated cells. MTT, 3-[4,5-dimethylthiazol-2-yl]-2,5-dimethyltetrazolium bromide.

We went on to investigate the gene expression levels of ERα in MCF-7/Fulv and MCF-7/Tam, as well as their proliferation capacity in the presence of estrogens. Higher mRNA levels of ERα were measured in MCF-7/Fulv and MCF-7/Tam (Fig. 3*A*). MCF-7/Fulv and MCF-7/Tam cells also expressed ERα protein as shown by immunoblotting (Supplemental Fig. S1_Revised; see https://doi.org/10.6084/m9.figshare.23667135.v1). Cell proliferation in MCF-7/Fulv and MCF-7/Tam was found to be independent of the presence of estrogens in different concentrations. Only the proliferation of parental MCF-7 cells showed a slight but significant increase in the presence of estradiol in concentrations of 1 nM and 10 nM (Fig. 3*B*). All the above advocate that hormone resistance in our cell lines is associated with the retention of ERα expression. Although ERα is present in MCF-7/Fulv and MCF-7/Tam, estradiol failed to induce their proliferation. It has been shown that ERα signaling activation may play a role in endocrine-resistant cell growth and is independent of the presence of estrogens. This is in agreement with the observations that the crosstalk between ERα and activated growth factor receptors, or their downstream kinases, in endocrine-resistant breast cancer cells supports the estrogen-independent activation of ERα (9).

### Development of Fulvestrant and Tamoxifen Resistance in MCF-7 Cells Is Associated with Activation of Tyrosine Kinase Receptors EGFR and HER

**Figure 4.**
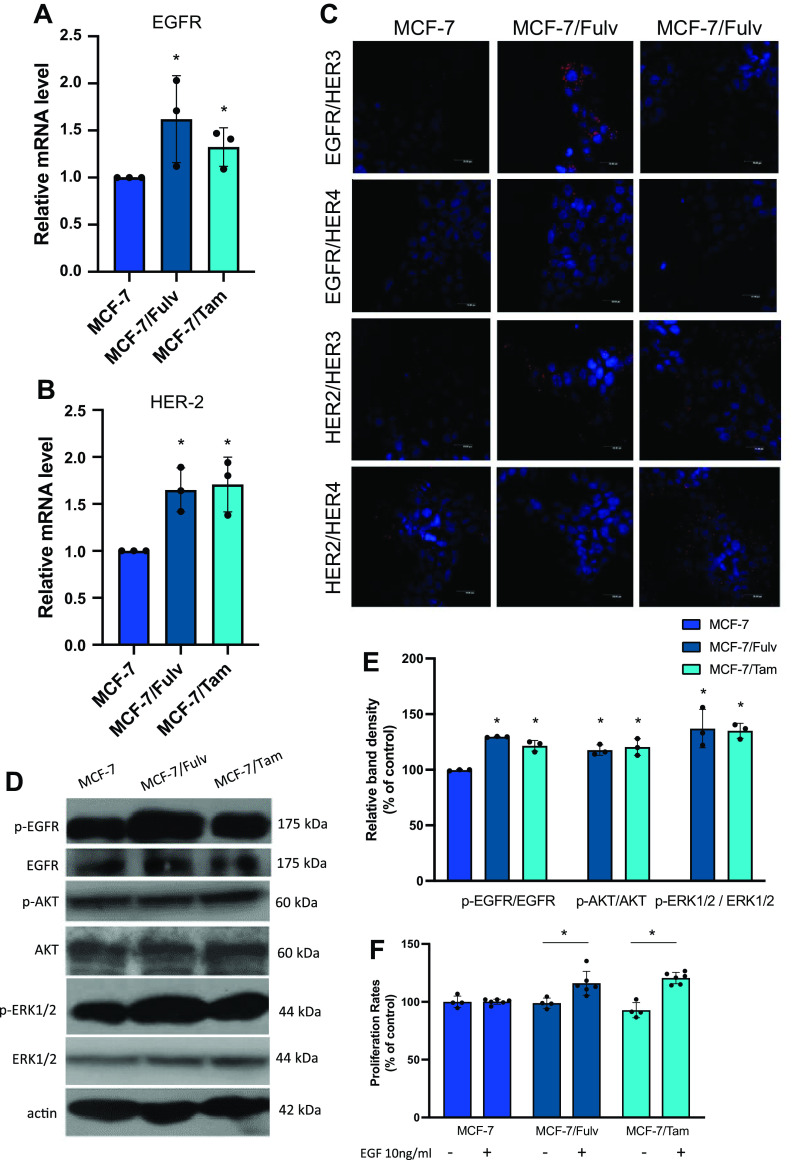
mRNA levels of epidermal growth factor receptor (EGFR; *A*) and human epidermal growth factor receptor 2 (HER2; *B*) in MCF-7, MCF-7 cells resistant to fulvestrant (MCF-7/Fulv), and MCF-7 cells resistant to tamoxifen (MCF-7/Tam) measured by RT-qPCR. Results are expressed as mean ± SD of relative mRNA level of three independent experiments. *Statistically significant differences (*P* ≤ 0.05) compared with parental MCF-7 cells. *C*: colocalization between members of EGFR/HER family receptors on MCF-7, MCF-7/Fulv, and MCF-7/Tam determined by proximity ligation assay. *D*: immunoblotting of pEGFR/EGFR, pAKT/AKT, pERK1/2/ERK1/2, and actin constitutively present in MCF-7, MCF-7/Fulv, and MCF-7/Tam cells. *E*: the ratio of phosphoEGFR (pEGFR) over EGFR, phosphoAKT (pAKT) over AKT, and phosphoERK1/2 (pERK1/2) over ERK1/2 was quantified from band density using ImageJ, and the values are displayed relative to the levels observed in the parental MCF-7 cells. Results are expressed as mean ± SD of % change of relative band density of three independent experiments. *Statistically significant differences (*P* ≤ 0.05) compared with parental MCF-7 cells. *F*: effect of epidermal growth factor (EGF) on cell proliferation in MCF-7, MCF-7 cells resistant to fulvestrant (MCF-7/Fulv), and MCF-7 cells resistant to tamoxifen (MCF-7/Tam) estimated by MTT assay. Cells were seeded in 48-well culture plates (10,000 cells/well) and cultured in RPMI without phenol red supplemented with 10% charcoal-stripped serum (CSS) in the presence or absence of EGF for 48 h. Results are expressed as mean ± SD of % change to control untreated cells of at least four independent experiments. *Statistically significant differences (*P* ≤ 0.05) compared with control untreated cells. MTT, 3-[4,5-dimethylthiazol-2-yl]-2,5-dimethyltetrazolium bromide.

Since mechanisms of crosstalk between ERα and tyrosine kinase receptors have been reported (33, 34), as well as the development of endocrine therapies resistance associated with the activation of receptor tyrosine kinases activation, we studied further the gene expression status of EGFR and HER2. Both MCF-7/Fulv and MCF-7/Tam were found to express increased mRNA levels for EGFR and HER2 (Fig. 4, *A* and *B*).

The increased gene expression levels of EGFR and HER2 led us to examine the colocalization of members of EGRF/HER family of tyrosine kinase receptors by proximity ligation assay. We found elevated levels of EGFR/HER3, EGFR/HER4, and HER2/HER3 heterodimers formed in MCF-7/Fulv compared with parental MCF-7 cells. MCF-7/Tam cells exhibited increased levels of HER2/HER3 and HER2/HER4 heterodimers compared with MCF-7 (Fig. 4*C*). In addition, EGFR protein expression levels in MCF-7/Fulv and MCF-7/Tam were defined by immunoblotting (Fig. 4*D*). Constitutively increased phosphorylation of EGFR (pEGFR), AKT (pAKT,) and ERK1/2 (pERK1/2) was found in both MCF-7/Fulv and MCF-7/Tam (Fig. 4, *D* and *E*). We studied further the effect of EGF on cell proliferation of the resistant cell lines, as EGFR is implicated in cell proliferation. The presence of EGF significantly induced cell proliferation only in resistant MCF-7/Fulv and MCF-7/Tam cells and not in parental MCF-7 cells (Fig. 4*F*).

**Figure 5.**
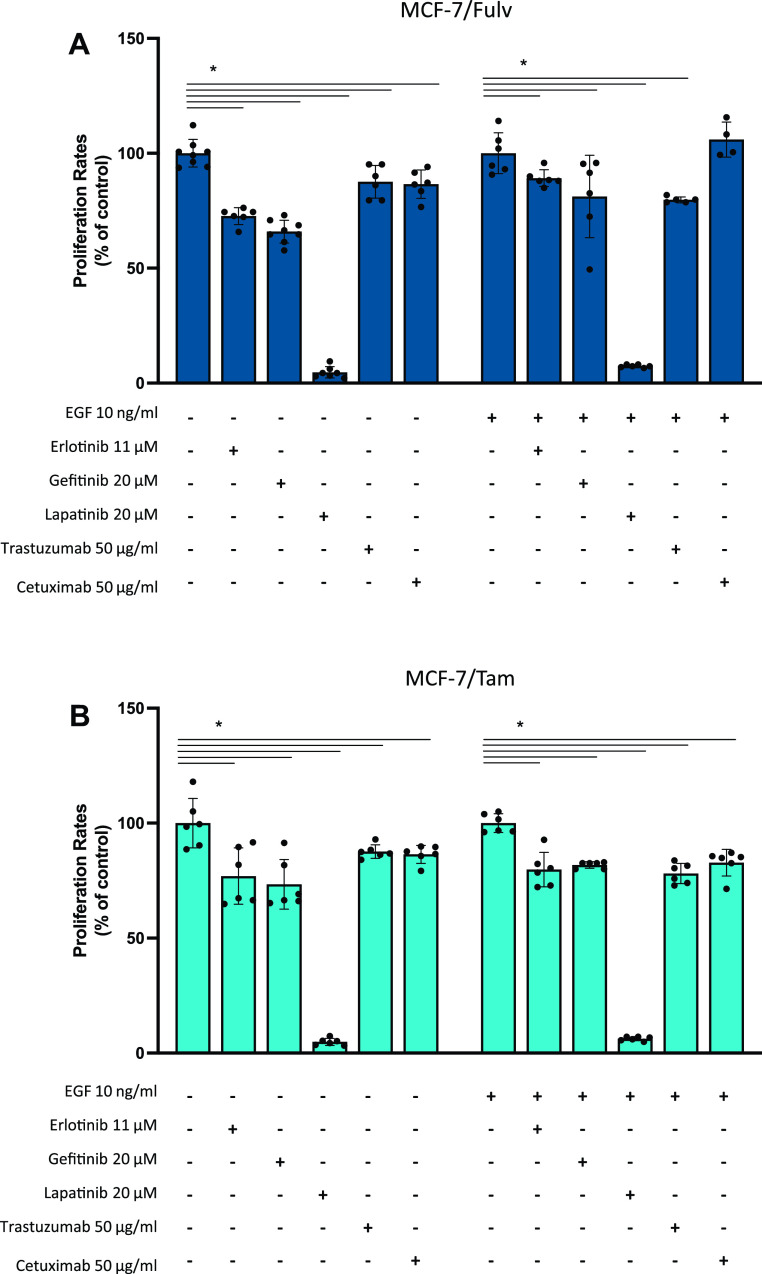
Effect of EGFR/HER signaling pathway inhibitors on MCF-7 cells resistant to fulvestrant (MCF-7/Fulv; *A*) and MCF-7 cells resistant to tamoxifen (MCF-7/Tam; *B*) cell proliferation. Cells were seeded in 48-well culture plates (10,000 cells/well) and cultured in RPMI without phenol red supplemented with 10% charcoal-stripped serum (CSS) in the presence or absence of epidermal growth factor (EGF) and various inhibitors for 48 h. Results are expressed as mean ± SD of % change to control untreated cells or cells treated only with EGF of at least four independent experiments. *Statistically significant differences (*P* ≤ 0.05) compared with control untreated cells. EGFR/HER, epidermal growth factor receptor and human epidermal growth factor receptor.

Given the fact that EGFR/HER signaling pathway is activated after resistance to hormone therapy, the effect of several EGFR/HER signaling pathway inhibitors, which are used in patients with metastatic breast cancer, on the proliferation of resistant MCF-7/Fulv and MCF-7/Tam cells was studied. Erlotinib, a tyrosine kinase inhibitor of EGFR phosphorylation; Gefitinib, a tyrosine kinase inhibitor of EGFR phosphorylation; Lapatinib, a dual tyrosine kinase inhibitor of HER2 and EGFR phosphorylation; Trastuzumab, recombinant humanized monoclonal antibody against HER2; and Cetuximab, chimeric monoclonal antibody against EGFR were applied in MCF-7/Fulv and MCF-7/Tam in the absence or the presence of EGF. Almost all tested inhibitors moderately reduced the proliferation rate in both MCF-7/Fulv and MCF-7/Tam (Fig. 5, *A* and *B*). In the case of coadministration of EGF, these agents exerted a lower inhibition capacity on breast cancer cell proliferation (Fig. 5, *A* and *B*). Among all tested agents, Lapatinib seemed to have a greater inhibitory effect in both MCF-7/Fulv and MCF-7/Tam cells. This might be because Lapatinib acts as a reversible inhibitor and targets both EGFR and HER2, indicating thus this agent is probably more effective in resistance to hormone therapy.

### The Development of Hormone Resistance Promotes Autophagy

**Figure 6.**
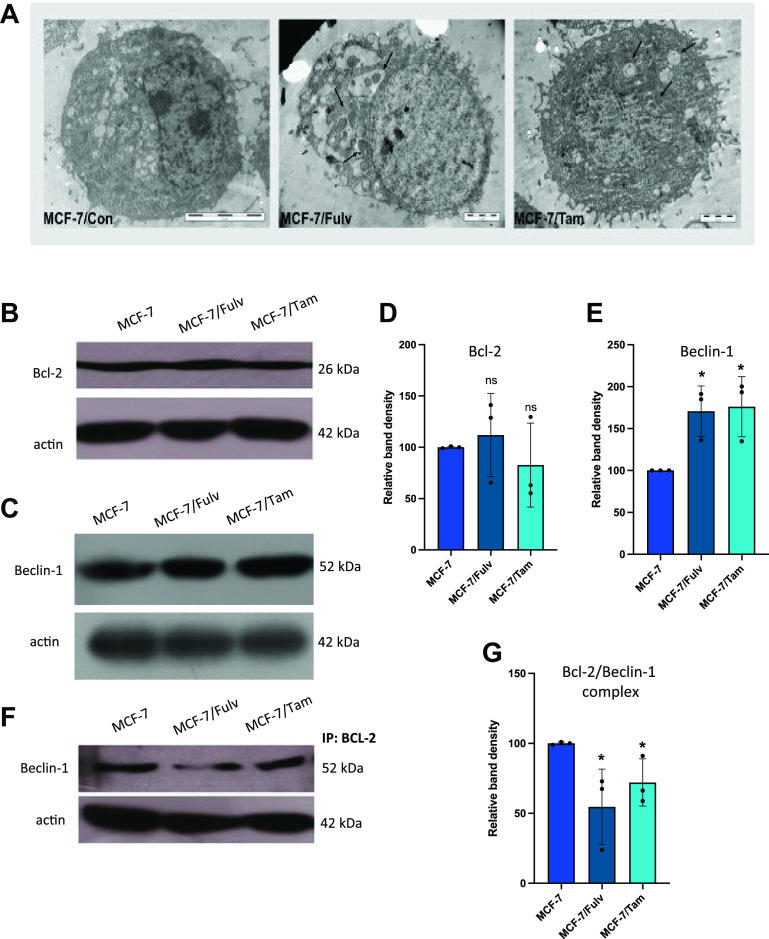
*A*: transmission electron microscopy (TEM) analysis indicating autophagosomes in parental MCF-7 cells and hormone-resistant MCF-7 cells resistant to fulvestrant (MCF-7/Fulv) and MCF-7 cells resistant to tamoxifen (MCF-7/Tam). Protein expression levels of Bcl-2 (*B* and *D*) and Beclin-1 (*C* and *E*) were determined by immunoblotting and quantified. Immunoprecipitation with antibody against Bcl-2 and identification of interacting Beclin-1 protein levels with immunoblotting (*F* and *G*). The density of immunoreactive bands was analyzed using ImageJ Software followed by normalization to the loading control (actin), and a percentage relative to the control MCF-7 cells was obtained. Results are expressed as mean ± SD of % change of relative band density of three independent experiments. *Statistically significant differences (*P* ≤ 0.05) compared with control parental MCF-7 cells.

Previous studies on breast cancer indicate a correlation between EGFR signaling pathways and autophagy (35). We studied the presence of autophagosomes by transmission electron microscopy (TEM) in parental and resistant MCF-7 cells. The existence of autophagosomes was revealed only in MCF-7/Fulv and MCF-7/Tam cells, indicating that resistance to hormone therapy possibly affects cells by inducing autophagy (Fig. 6*A*). We further investigated the induction of autophagy in MCF-7/Fulv and MCF-7/Tam cells by measuring protein expression levels of Beclin-1, a protein that is tightly related to autophagy (36). In addition to that, Bcl-2, an antiapoptotic protein, was also examined as this protein interacts with Beclin-1 forming complexes and blocks Beclin-1 to induce autophagy (37). No significant differences were observed in Bcl-2 protein levels between resistant cell lines and the parental one (Fig. 6, *B* and *D*). On the contrary, Beclin-1 protein levels were increased in both MCF-7/Fulv and MCF-7/Tam (Fig. 6, *C* and *E*). Increased protein levels of Beclin-1 are related to worse overall survival outcomes either in patients with ERα/PR-positive/HER2-negative breast cancer or in patients with breast cancer independent of their ERα/PR/HER2 status [Supplemental Fig. S1_Revised; see https://doi.org/10.6084/m9.figshare.23667135.v1, https://kmplot.com/analysis/ (38)], suggesting the association of Beclin-1 and most likely increased autophagy with breast cancer cell aggressiveness. The interaction of Bcl-2 with Beclin-1 was evaluated by immunoprecipitation. As shown in Figure 6, *F* and *G*, lower levels of Beclin-1 are involved in the formation of Bcl-2/Beclin-1 complexes in both MCF-7/Fulv and MCF-7/Tam, implying that greater levels of free Beclin-1 are present following resistance, to promote autophagy. Finally, an apoptosis detection assay was performed by flow cytometry in parental and hormone therapy-resistant MCF-7 cells in the presence and absence of estrogens. No differences in apoptosis were observed between parental and resistant cell lines (Supplemental Fig. S1_Revised; see https://doi.org/10.6084/m9.figshare.23667135.v1). These results suggest that the activation of autophagy in hormone-resistant MCF-7/Fulv and MCF-7/Tam may provide cells with a significant advantage that enables cells’ survival.

## DISCUSSION

Resistance to endocrine therapy remains a major problem that must be confronted in clinical practice. Acquired hormone therapy resistance underlies various mechanisms that remain unclear and have to be explored. In the current study, we have established MCF-7 breast cancer cell lines resistant to hormone therapy consisting of Fulv that belongs to SERDs and Tam that acts as SERM (6). MCF-7 cells are considered to have low clonogenic potential and be less aggressive compared with other breast cancer cell lines (39). Interestingly, the established cell lines resistant to hormone therapy, exert increased colony formation ability in soft agar, implying their augmented capacity for survival and growth promotion (40). Resistant cells established by our group seem to have developed mechanisms to survive and proliferate in a 3-D environment to mimic the tumor microenvironment in the human body. In contrast to 3-D cultures, resistant cell lines proliferate at lower rates in 2-D cultures. This phenomenon might take place due to lower metabolic processes in the cells. Perhaps, the resistant cells try to retain energy to provide it for cell motility and migration as well as survival in a stressful environment as previously shown in tyrosine kinase inhibitor-resistant glioblastoma cells (31).

In our study, resistance both to SERDs and SERMs results in the retention of ERα expression but renders cells independent of estrogens for their survival and growth. These results are in accordance with previous studies concerning acquired drug resistance that refer to ERα signaling without its ligand binding (41). Various mechanisms have been proposed to explain ERα activity. The mutant estrogen receptor is considered one of the prevalent opinions that triggers signaling in the absence of estrogens. Another mechanism related to the development of endocrine resistance is the crosstalk between ERα and tyrosine kinase receptors like EGFR and HER2 (33, 42, 43).

Apart from *ERα* gene (*ESR1*) mutations, endocrine resistance in advanced breast cancer is also associated with activating *HER2* and loss of function *NF1* mutations, alterations in other mitogen-activated protein kinase (MAPK) pathway genes including *EGFR*, *KRAS*, *HRAS*, *BRAF*, and *MAP2K1* (*MEK1*), as well as ER transcriptional regulators such as *MYC*, *CTCF*, *FOXA1*, and *TBX3* (44). Razavi et al. (44) reported that these alterations were mutually exclusive with *ESR1* mutations. Alterations in effectors of MAPK signaling are related to shorter progression-free survival on AI or SERD therapies (44). Clinical evidence has shown that resistance in breast cancers triggers the expression of tyrosine kinase receptors like ERBB1/EGFR and other members of this family as ERBB2/HER2 (45, 46). It has been shown that *ERBB2/HER2*-amplified tumors are resistant to hormonal therapy (47–50). Indeed, in the current study not only the overexpression of EGFR and HER2 from MCF-7/Fulv and MCF-7/Tam is observed, but also increased levels of heterodimers such as EGFR/HER3, EGFR/HER4, and HER2/HER3 in MCF-7Fulv and HER2/HER3 and HER2/HER4 in MCF-7Tam are detected. Heterodimerization of ERBB family receptors is related to breast cancer aggressiveness. For example, heterodimerization of HER2 with either HER3, HER4, or EGFR has been associated with breast cancer cell growth, treatment resistance in preclinical studies, and worse outcomes in patients with ER^+^ breast cancer (51). Furthermore, in our study, the development of resistance both to SERDs and SERMs is accompanied by the activation of EGFR/HER2 downstream pathways such as mitogen-activated protein kinase (MAPK)/ERK1/2 as well as PI3K/AKT. This is in accordance with a previous study showed that Tam-resistant MCF-7 cells exhibit increased levels of EGFR/HER2, phosphorylation of EGFR/HER2 and EGFR/HER3 heterodimers, and activation of ERK1/2 (52). MAPK signaling cascade can be triggered either by EGFR and/or HER2 ending up in phosphorylation of ERα serine 118 or 167 (53, 54). It has been shown that the overexpression of EGFR in MCF-7 cells provides cancer cells with resistance to Fulv, and the resistant phenotype can be reversed upon cotreatment with a combination of Fulv and the EGFR inhibitors, erlotinib or gefitinib (44). Notably, selective targeting of MAPK signaling with ERK inhibitors can resensitize EGFR overexpressing MCF-7 to Fulv (44). Upregulated EGFR/MEK1/MAPK1/2 signaling also occurs in 4-hydroxytamoxifen-resistant MCF-7 breast cancer cells and blocks BimEL-dependent apoptosis (55). The use of tyrosine kinase inhibitors has been previously described in some cases as a possible option to overcome hormone resistance (52, 56–59). Our data clarified that the best inhibitory effect on the proliferation of hormone therapy-resistant MCF-7/Fulv and MCF-7/Tam cells was observed in the case of double inhibition of both EGFR and HER2 by using lapatinib.

EGFR/HER2 signaling pathway has a crucial role in acquired endocrine resistance, and the exact mechanisms that underlie merit further investigation. For example, both EGFR/HER2 apart from their ability to activate oncogenic signaling can also regulate autophagy, which is involved in the development of hormone-therapy resistance (60–62). Both EGFR/HER2 signaling and autophagy cooperate during the development of endocrine resistance. Autophagy is adopted by cells in cases of nutrient deprivation, stress factors, or damaged organelles and proteins. It can either lead to cell survival or cell death (63), and there is evidential interaction between the autophagic protein Beclin-1 and the antiapoptotic protein Bcl-2 (64). In addition, it has been suggested that metastatic cancer cells may escape from anoikis via the induction of autophagy (65, 66). EGFR is a crucial regulator of autophagy exhibiting a dual activity. In nutrient‐rich growth conditions, ligand‐activated EGFR inhibits autophagy either through phosphorylation of Beclin-1 or by activation of AKT/mTORC1 pathway (61). On the other hand, under serum‐starved conditions, ligand‐unbound EGFR, which constitutively traffic toward the endosomes and increases in the endosomal pool, interacts with the autophagy inhibitor Rubicon promoting its dissociation from Beclin-1 permitting Beclin-1 activation and induction of autophagy (67). Beclin-1 directly interacts with HER2 at the cell surface and is involved in HER2 and AKT phosphorylation (68). It has been shown that HER2-expressing breast cancer cells that are resistant to lapatinib exhibit increased cytoprotective autophagy. Lapatinib disrupts the cell surface interaction between HER2 and Beclin-1 and subsequently increases the cytosolic levels of Beclin-1 that in turn can induce autophagy (68).

In our study, autophagy is activated in hormone therapy-resistant MCF-7/Fulv and MCF-7/Tam cells, and it may contribute to cells’ survival and aggressive phenotype. In MCF-7/Fulv and MCF-7/Tam cells, increased expression of Beclin-1, as well as a higher percentage of dissociated Beclin-1 from Bcl-2 complex, was found, and this might have a dual role: elevated levels of free Beclin-1 can induce autophagy, whereas increased levels of free Bcl-2 protein are able to exert a potent antiapoptotic role. This is in accordance with the presence of autophagosomes in resistant cells and the lack of apoptosis, although cells are cultured in the presence of Tam and Fulv. The induced autophagy may cooperate with activated EGFR/HER2 signaling not only to develop endocrine resistance but also to drive cell-aggressive phenotype. Further studies to clarify the crosstalk of these pathways and the molecular mechanisms underlying the cooperative action of autophagy and EGFR/HER2 signaling to regulate breast cancer cells’ endocrine resistance and phenotype are required. Previous studies have proposed autophagy as a mechanism for 4-hydroxytamoxifen resistance (69–71). The 4-hydroxytamoxifen-resistant MCF-7 breast cancer cells can survive EGFR targeting by activating pro-survival autophagy (55). The induction of autophagy is responsible for the development of resistance to Tam in breast cancer cells (72, 73). The acquired resistance to Tam is related to elevated hexokinase II expression and elevated glycolysis rate. Hexokinase II interacts with mTOR and inhibits mTOR-S6K signaling to promote autophagy (72). In addition, metastasis-associated 1 protein, which has been implicated in breast tumorigenesis and metastasis, is upregulated in Tam-resistant breast cancer cells. It induces AMPK activation and subsequently autophagy that contribute to the development of Tam resistance in breast cancer (73). It has been also shown that autophagy fosters aggressive phenotype of cancer cells by facilitating anoikis resistance and lung colonization (74) as well as by inducing epithelial to mesenchymal transition (EMT), cancer cell growth, and metastasis (75–78). So, cytoprotective autophagy emerges as a novel resistance mechanism to endocrine (11, 72) and anti-HER2 therapies (62, 79, 80). Preclinical studies support this notion since trastuzumab- or lapatinib-refractory HER2 breast cancer cells exhibit increased formation of autophagosomes, which were essential for their survival (79, 80).

Autophagy is also regulated by PI3K/AKT/mTOR signaling axis as well as RAS/RAF/ERK pathway (35, 81, 82). Aberrant activation of ERK can promote autophagy in certain cell types (81). Activation of PI3K/AKT/mTOR axis in cancer cells not only suppresses autophagy but also induces protein translation, cell growth, and proliferation to drive tumorigenesis. On the other hand, tumors with elevated metabolic demands, with either constitutively active PI3K mutations, or AKT activation, may be dependent on autophagy for energy homeostasis and survival. Increased autophagy is required in Kras-driven tumor cells to provide amino acids, such as glutamine or glutamate, to maintain energy charge and nucleotide pools necessary for their survival (83). In addition, tumor cells engage surrounding stromal cells as active and essential microenvironmental contributors of nutrients by activating stromal cells’ autophagy to support tumor cell growth and invasion (84). Defective autophagy induced by PI3K/AKT/mTOR signaling activation might have negative implications on the survival of rapidly proliferating tumor cells, so compensatory mechanisms might be activated to counterbalance the suppression of autophagy by mTOR activation. So, acquired resistance to hormone therapy and overexpression of EGFR/HER2 and activation of EGFR/HER2/MAPK pathway may counterbalance the mTOR activation by PI3K/AKT providing breast cancer cells with potent oncogenic signaling and high levels of cytoprotective autophagy.

### Conclusions

Our data demonstrate that resistance to hormone therapy caused by both Fulv and Tam promotes autophagy with concomitant apoptosis evasion, rendering cells capable of surviving and growing. The fact that resistance also triggers ERBB family signaling pathways, which are poorly inhibited by tyrosine kinase inhibitors, might attribute to cells a more aggressive phenotype. It is obvious that the development of endocrine therapy resistance involves a complex interplay between deregulated ERBB signaling and autophagy that may be considered in clinical practice. Novel therapeutic interventions such as EGFR/HER2/MAPK pathway and autophagy inhibitors could be tested in combination with hormonal therapy to prevent resistance in hormone-dependent breast cancer.

## DATA AVAILABILITY

The authors declare that the data supporting the findings of this study are available within the article.

## SUPPLEMENTAL DATA

10.6084/m9.figshare.23667135.v1 Supplemental Fig. S1_Revised: https://doi.org/10.6084/m9.figshare.23667135.v1.

## GRANTS

This research was funded by Princess Nourah bint Abdulrahman University Researchers Supporting Project number (PNURSP-HC2022/6) to A.D.T. and F.M.A., Princess Nourah bint Abdulrahman University, Riyadh, Saudi Arabia.

## DISCLOSURES

No conflicts of interest, financial or otherwise, are declared by the authors.

## AUTHOR CONTRIBUTIONS

E.G., H.P.K., and A.D.T. conceived and designed research; K.E.S., E.G., P.S., M.V.K., S.R., and K.F. performed experiments; K.E.S., E.G., D.M., P.S., M.V.K., S.R., K.F., and F.M.A. analyzed data; K.E.S., E.G., D.M., H.P.K., and A.D.T. interpreted results of experiments; K.E.S., D.M., and F.M.A. prepared figures; K.E.S., E.G., D.M., F.M.A., and A.D.T. drafted manuscript; H.P.K. and A.D.T. edited and revised manuscript; K.E.S., E.G., D.M., P.S., M.V.K., S.R., K.F., F.M.A., H.P.K., and A.D.T. approved final version of manuscript.
